# Bilateral Lower Extremity Necrotizing Fasciitis Caused by Extended-Spectrum Beta-Lactamase-Producing Escherichia coli in a Liver Transplant Recipient

**DOI:** 10.7759/cureus.106649

**Published:** 2026-04-08

**Authors:** Natsumi Naka, Yuto Yamamura, Kazuyasu Fujii, Chisa Nakashima, Atsushi Otsuka

**Affiliations:** 1 Dermatology, Kindai University Hospital, Osaka, JPN

**Keywords:** bilateral involvement, esbl-producing escherichia coli, immunocompromised host, liver transplantation, necrotizing fasciitis

## Abstract

Necrotizing fasciitis (NF) is a rapidly progressive and life-threatening soft tissue infection that typically affects a single extremity, whereas bilateral or multifocal involvement is rare and associated with high mortality. Gram-negative organisms, including extended-spectrum beta-lactamase (ESBL)-producing Escherichia coli, are uncommon causes but are increasingly recognized in immunocompromised patients and are often associated with fulminant clinical courses.

We report a rare case of bilateral lower extremity NF caused by ESBL-producing E. coli in a woman in her seventies with a history of liver transplantation under long-term immunosuppression. The patient initially presented with bilateral lower leg erythema and swelling without necrosis, leading to a preliminary diagnosis of cellulitis. Despite antimicrobial therapy, her condition deteriorated with the development of systemic inflammatory response, coagulopathy, and subsequent localized necrosis. Imaging revealed subcutaneous gas, and surgical exploration confirmed NF. Both blood and wound cultures grew ESBL-producing E. coli, prompting escalation to carbapenem therapy. Repeated surgical debridement was performed, resulting in eventual infection control and successful limb preservation.

This case highlights several important clinical considerations. First, NF caused by ESBL-producing E. coli may present with subtle early cutaneous findings despite rapid systemic deterioration, particularly in immunocompromised hosts. Second, bilateral involvement, although rare, should be considered in patients with risk factors such as chronic lymphedema and bacteremia. Finally, early recognition, prompt surgical exploration, and aggressive debridement combined with appropriate antimicrobial therapy are critical for survival and limb preservation.

## Introduction

Necrotizing fasciitis (NF) is a rapidly progressive, life-threatening soft tissue infection that requires prompt diagnosis and aggressive surgical intervention [1]. NF usually involves a single extremity, and bilateral or multifocal presentations are rare [2,3]. Such cases are associated with delayed recognition, higher morbidity, and increased mortality. Well-recognized predisposing factors include diabetes mellitus, chronic kidney disease, liver dysfunction, malignancy, and immunosuppression [1]. In particular, solid organ transplant recipients on long-term immunosuppressive therapy are highly vulnerable, and fatal cases of Escherichia coli (E. coli) NF have been reported in this population [4,5].

Although Gram-negative bacilli are less common causes of NF than Group A streptococci or staphylococci, their frequency appears to be increasing in immunocompromised hosts [6]. Among them, ESBL-producing E. coli pose a particular therapeutic challenge [5]. In contrast to typical NF caused by Staphylococcus aureus or other common pathogens, Gram-negative NF-particularly that due to ESBL-producing E. coli-is characterized by more fulminant progression, early systemic toxicity, and sometimes deceptively subtle early cutaneous findings [1,6,7]. These infections are associated with higher rates of sepsis, organ dysfunction, and mortality compared with NF caused by usual pathogens.

Herein, we report a rare case of bilateral lower extremity NF caused by ESBL-producing E. coli in a liver transplant recipient under long-term immunosuppression. This case highlights the importance of maintaining a high index of suspicion for atypical infections in immunocompromised patients and the critical role of timely surgical intervention.

## Case presentation

A 70-year-old woman with a complex medical history was referred to our department for evaluation of bilateral lower leg erythema, swelling, and fever, which had persisted for four days before referral. Her past history included Guillain-Barré syndrome and primary biliary cholangitis, for which she had previously undergone living donor liver transplantation and remained on long-term immunosuppression with prednisolone and tacrolimus. She also had a history of endometrial cancer treated surgically more than a decade earlier, complicated by chronic bilateral lower limb lymphedema. Several months before presentation, she sustained multiple fractures in a traffic accident and was hospitalized for conservative treatment and rehabilitation.

**Figure 1:**
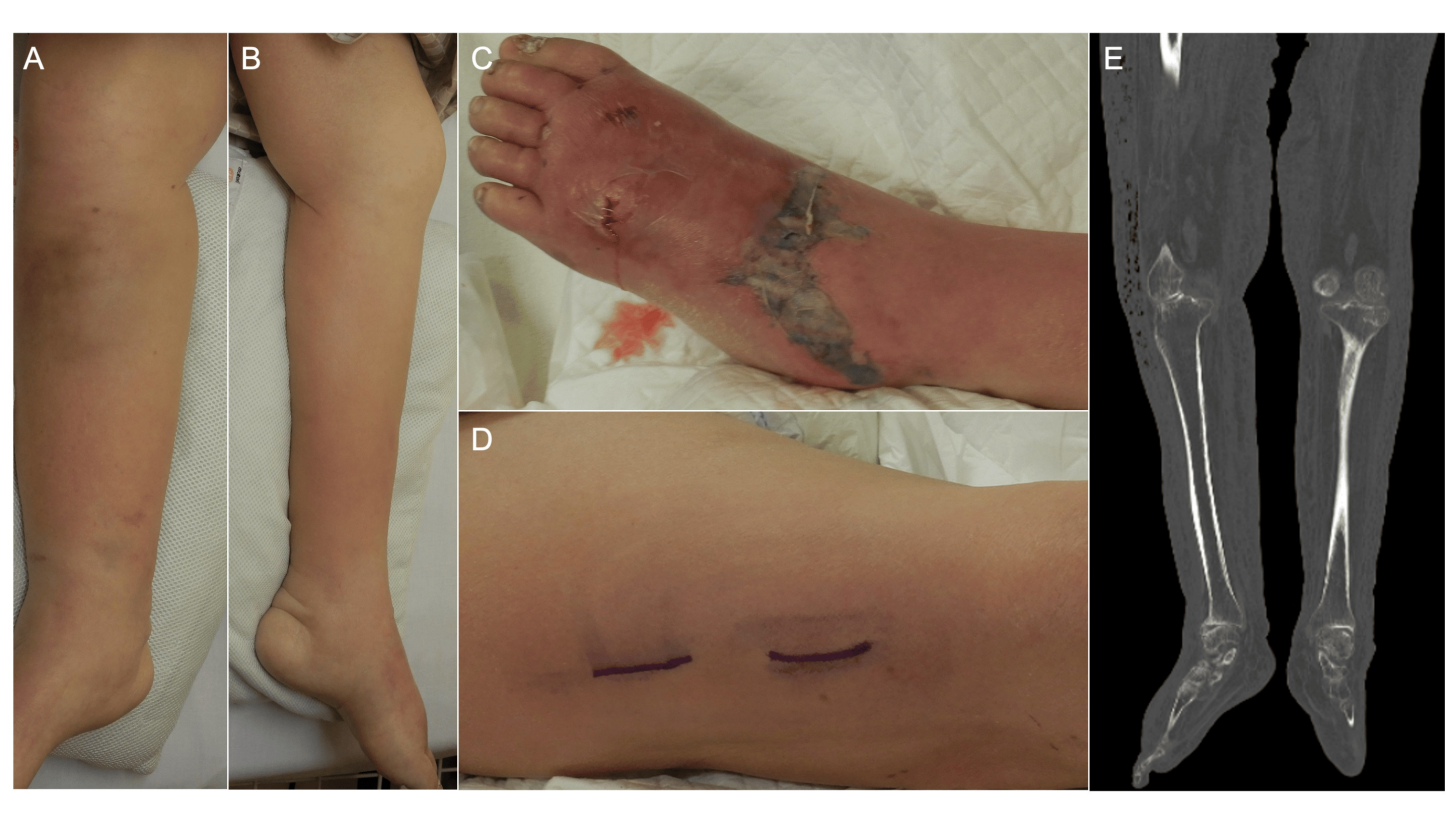
Clinical and radiologic findings during the early disease course. (A, B) Clinical appearance of the right lower leg (A) and left lower leg (B) on admission, showing erythema and swelling without necrosis. (C) Left foot on hospital day three, showing newly developed dorsal skin necrosis. (D) Right lateral thigh on hospital day three, exhibiting erythema without necrosis or purpura. The dark purple markings indicate planned incision lines. (E) Computed tomography on hospital day three demonstrating scattered subcutaneous gas foci distributed from the distal thigh to the proximal lower leg, spanning the knee joint (outlined).

During rehabilitation, she had developed bilateral lower leg erythema and swelling in the absence of any necrotic or purpuric changes. Oral cefcapene pivoxil was administered but failed to produce clinical improvement, leading to hospital referral. On admission, physical examination again showed bilateral erythema and swelling of the lower legs consistent with cellulitis, without any necrotic changes or purpuric lesions (Fig 1A, 1B). Laboratory tests showed marked inflammation and coagulation abnormalities (Table 1): CRP 39 mg/dL, WBC 2,710/µL, platelets 59,000/µL, creatinine 2.43 mg/dL, fibrin degradation products (FDP) 50 µg/mL, and markedly elevated procalcitonin (98 ng/mL).

**Table 1:** Laboratory findings on admission. Abbreviations: CRP: C-reactive protein; WBC: White blood cell count; FDP: Fibrin degradation products; BUN: Blood urea nitrogen; PT-INR: Prothrombin Time/International Normalized Ratio

Test	Value	Reference range	Interpretation
CRP	39 mg/dL	<0.3 mg/dL	markedly elevated
WBC	2,710 /µL	3500–9000 /µL	decreased
Hemoglobin	8.0 g/dL	12.0–15.0 g/dL	decreased
Platelet	59,000 /µL	150000–350000 /µL	decreased
Creatinine	2.43 mg/dL	0.6–1.1 mg/dL	elevated
BUN	62 mg/dL	8–20 mg/dL	elevated
FDP	50 µg/mL	<5 µg/mL	markedly elevated
Procalcitonin	98 ng/mL	<0.05 ng/mL	markedly elevated
PT-INR	0.99	0.9–1.1	within normal limits

She was diagnosed with sepsis and disseminated intravascular coagulation secondary to bilateral lower-extremity cellulitis. Empiric antimicrobial therapy with cefmetazole (1 g twice daily), teicoplanin (400 mg twice daily), and micafungin (100 mg once daily) was initiated.

After admission, new-onset skin necrosis developed on the dorsum of the left foot (Fig 1C). The right leg showed only swelling and erythema without overt necrosis (Fig 1D); however, computed tomography demonstrated scattered subcutaneous gas foci extending from the thigh across the knee into the proximal lower leg (Fig 1E). Exploratory incision revealed necrotic tissue, and the finger test was positive, confirming the diagnosis of necrotizing fasciitis. Wound cultures obtained during the procedure and blood cultures drawn on admission both yielded ESBL-producing Escherichia coli. Specimens obtained during the procedure were submitted for both aerobic and anaerobic cultures; however, no anaerobic organisms were isolated. Antimicrobial therapy was therefore switched to doripenem (0.5 g twice daily). Doripenem was continued as intravenous therapy until day 42, with no transition to oral antimicrobial agents during this period. Progression of tissue necrosis necessitated repeated debridement, including the right thigh, right lower leg, left lower leg, and dorsal left foot.

With continued treatment, blood cultures became negative by day six, whereas wound cultures remained positive until day 27, after which inflammatory markers gradually improved. Multiple cerebral infarctions complicated her clinical course due to new-onset atrial fibrillation and bleeding from the wound sites during continuous hemodiafiltration, but her overall condition eventually stabilized. She was transferred to a general ward on day 20. On day 42, split-thickness skin grafting was performed over the fasciotomy and debridement sites. Approximately three months after admission, the grafts had healed well, allowing preservation of both lower limbs.

## Discussion

This case is notable not only because it presented bilaterally but also because it was caused by ESBL-producing E. coli and showed only subtle early cutaneous findings in an immunosuppressed host. Multifocal (non-contiguous) NF is considered extremely rare, with only scattered case reports identified in systematic reviews [8]. In the present case, the lesions were bilateral but non-contiguous. Previously reported cases of bilateral NF or NF caused by ESBL-producing E. coli have often been associated with death or major limb loss, making the present case notable for survival with preservation of both lower limbs [3,6]. Compared with NF caused by Staphylococcus aureus or other usual pathogens, ESBL-producing E. coli NF tends to progress more rapidly, cause earlier systemic toxicity, and exhibit higher mortality [1,6,7]. Importantly, early cutaneous manifestations may be minimal.

In solid organ transplant recipients, chronic immunosuppression substantially increases both the risk and severity of necrotizing infections and may blunt early inflammatory signs [4,6]. In our patient, long-term immunosuppression following liver transplantation, together with chronic lower limb lymphedema, likely contributed to both the development of NF and the deceptively mild initial cutaneous findings. Early bacteremia combined with bilateral chronic lymphedema may have contributed to the bilateral distribution of necrotizing fasciitis. Several studies have noted that Gram-negative NF, including infections caused by ESBL-producing E. coli, may initially present with nonspecific erythema or edema, with necrosis developing only later in the disease course [1,6,7]. This pattern can make early clinical recognition particularly challenging, especially in immunocompromised hosts.

The causative organism was ESBL-producing E. coli. Gram-negative NF is rare, but when present, it is strongly associated with bacteremia and septic shock [7]. In our patient, both wound and blood cultures yielded ESBL-producing E. coli early in the clinical course, confirming bacteremia at presentation. ESBL-producing strains further limit therapeutic options, necessitating prompt carbapenem therapy and aggressive debridement. In the present case, identification of ESBL-producing E. coli from wound cultures prompted escalation to doripenem, in combination with repeated debridement, which was likely critical for both survival and limb preservation.

Early diagnosis of NF is challenging because early cutaneous signs may initially be subtle or even indistinguishable from non-necrotizing infections [1]. In our patient, systemic deterioration despite initially stable-appearing local findings prompted exploratory incisions, which confirmed NF bilaterally. This underscores current recommendations that a low threshold for surgical exploration and use of the “finger test” is crucial when NF is suspected.

## Conclusions

This case illustrates bilateral NF of the lower extremities caused by ESBL-producing E. coli in a liver transplant recipient under long-term immunosuppression, with a favorable outcome of both survival and limb preservation. While bilateral NF is exceedingly rare and often fatal, our case highlights two broadly applicable lessons: clinicians should maintain vigilance for atypical or multifocal NF in immunocompromised patients, and decisive early interventions-including exploratory incisions, finger testing, and repeated aggressive debridement-are universally critical in the management of severe NF. Because dermatologists are often the first to evaluate patients presenting with bilateral erythema or cellulitis-like manifestations, heightened awareness of atypical or multifocal NF is particularly important in dermatologic practice. In addition, because early cutaneous signs in ESBL-producing E. coli NF may be deceptively mild, dermatologists should be particularly alert to rapid systemic deterioration disproportionate to skin findings-a key diagnostic clue emphasized in pathogen-comparative studies.
